# Laboratory diagnosis of 44 cases of pediatric histiocytic necrotizing lymphadenitis

**DOI:** 10.3389/fped.2025.1638239

**Published:** 2025-08-28

**Authors:** Jingjing Jin, Songjian Lu, Honghua Lin

**Affiliations:** Department of Clinical Immunology, Anhui Provincial Children’s Hospital, Hefei, China

**Keywords:** histiocytic necrotizing lymphadenitis, Kikuchi-Fujimoto disease, children, prognosis, laboratory diagnosis

## Abstract

**Objective:**

To investigate the clinical features, laboratory findings, treatment, and follow-up of pediatric histiocytic necrotizing lymphadenitis (HNL) to enhance understanding of this disease.

**Methods:**

A retrospective analysis was conducted on clinical data of 44 pediatric patients diagnosed with histiocytic necrotizing lymphadenitis (HNL) who were admitted to our hospital from January 2018 to October 2024. The clinical features, laboratory findings, pathological results, treatment, and prognosis were examined.

**Results:**

44 patients, aged 4–14 years (mean age: 9.4 ± 2.6 years), including 28 males and 16 females, Among them, 41 patients (93.2%) were aged ≥6 years. All patients presented with fever and superficial lymphadenopathy. Other clinical manifestations comprised rash (10 cases, 22.7%), abdominal pain and vomiting (7 cases, 15.9%), hepatosplenomegaly (5 cases, 11.4%), weight loss (3 cases, 6.8%), and fatigue (3 cases, 6.8%). The clinical manifestations of HNL (e.g., fever, cervical lymphadenopathy) were nonspecific and overlapped with other pediatric conditions. Definitive diagnosis required lymph node biopsy, which consistently demonstrated HNL in all cases. Laboratory findings predominantly showed normal or reduced leukocyte counts (42 cases, 95.5%), elevated lactate dehydrogenase (LDH) (38 cases, 86.4%), increased erythrocyte sedimentation rate (ESR) (34 cases, 77.3%), and elevated D-dimer levels (28 cases, 63.6%). Lymph node ultrasound (36 cases, 81.8%) revealed hypoechoic nodules, while neck CT (7 cases, 15.9%) demonstrated nodular soft-tissue density shadows. Glucocorticoids were administered to 35 cases (79.5%). Two cases (4.5%) of secondary hemophagocytic lymphohistiocytosis (HLH) were treated with methylprednisolone pulse therapy or intravenous immunoglobulin. Three cases (6.8%) were administered glucocorticoids combined with disease-modifying antirheumatic drugs, 1 case (2.3%) received nonsteroidal anti-inflammatory drugs (NSAIDs) alone, and 8 cases (18.2%) resolved spontaneously without intervention. During a follow-up period ranging from 2 months to 6 years, no cases progressed to other rheumatic diseases, 5 cases (11.4%) experienced recurrence, whereas the other cases exhibited a satisfactory prognosis.

**Conclusion:**

The clinical manifestations of HNL in pediatric patients are nonspecific, necessitating lymph node biopsy for definitive diagnosis. It is glucocorticoid-sensitive, and some cases may resolve spontaneously with a positive prognosis, but long-term monitoring is essential.

## 1 Introduction

Histiocytic necrotizing lymphadenitis (HNL), also known as Kikuchi-Fujimoto disease (KFD) or subacute necrotizing lymphadenitis (SNL), is a benign, self-limited disorder marked by fever, superficial lymphadenopathy, and normal or reduced leukocyte counts (1–3). First reported by Kikuchi and Fujimoto in 1972, HNL predominantly affects adults. While HNL has been widely reported in adults, studies focusing on its pediatric presentation remain limited, particularly in the Chinese population. The nonspecific nature of fever and superficial lymphadenopathy might lead to misdiagnosis or delays in diagnosis and treatment. The diagnosis, treatment, and prognosis of HNL markedly differ from those of lymphoma and systemic lupus erythematosus. This article retrospectively examines the clinical data, laboratory results, and prognosis of 44 pediatric cases of HNL identified and treated at our hospital to enhance understanding of this disease.

## 2 Subjects and methods

### 2.1 Study population

A retrospective analysis was performed on 44 pediatric patients with HNL identified through lymph node biopsy at our hospital from January 2018 to October 2024.This study was approved by the Medical Ethics Committee of Anhui Provincial Children's Hospital (Approval No. EYLL-2023-041) and was conducted in accordance with the principles of the Declaration of Helsinki. Written informed consent was obtained from all patients' legal guardians.

### 2.2 Method

1. Data Collection: Clinical data included demographics (name, sex, age), clinical manifestations (triggers, disease course, fever characteristics, lymphadenopathy, rash, arthralgia, abdominal pain, hepatosplenomegaly), laboratory tests (complete blood count, serum ferritin, C-reactive protein, autoantibodies, ESR, liver or kidney function, pathogen testing, Lactate Dehydrogenase (LDH), immune function, coagulation function), imaging studies (lymph node ultrasound, neck computed tomography), bone marrow aspiration, lymph node biopsy pathology, and treatment outcomes.
2. Follow-up: Patients were observed through outpatient or inpatient visits until December 2024.
3. Statistical Methods: Data were processed using Microsoft Excel software. Categorical data are expressed as frequencies (*n*) and percentages (%), whereas continuous data are denoted as mean ± standard deviation (*x* ± *s*).

## 3 Results

### 3.1 General characteristics

Of the 44 patients, 28 were male and 16 were female. Forty-one patients (93.2%) were between the ages of 6 and 14 years (28 males, 13 females). The age of onset ranged from 4 to 14 years, with a mean of 9.4 ± 2.6 years.

### 3.2 Clinical manifestations

All 44 patients presented with fever and superficial lymphadenopathy at disease onset. The fever pattern was irregular, and the average time between the onset of early symptoms and the conclusive diagnosis of HNL was 20.5 ± 9.9 days (range: 8–60 days). Twenty-six patients (59.1%) initially exhibited fever, 8 (18.2%) came with cervical lymphadenopathy, and 10 (22.7%) with concurrent fever and lymphadenopathy at onset. Cervical lymphadenopathy was present in all 44 cases (100%), Cervical lymphadenopathy predominated in 31 cases (70.5%), with bilateral cervical involvement in 22 cases (50.0%). Thirteen patients (29.5%) had lymphadenopathy in other regions, such as supraclavicular, inguinal, axillary, and popliteal lymph nodes. The average lymph node size was 24.4 ± 6.4 mm (range: 10–41 mm). Tenderness was observed in the lymph nodes of 30 patients (68.2%), while none of the 44 patients displayed erythema or ulceration on the surface of superficial lymph nodes. The consistency of lymph nodes was heterogeneous: firm in 26 patients (59.1%), soft in 10 (22.7%), and moderate in 8 (18.2%). Other manifestations comprised rash (10 cases, 22.7%), abdominal pain and vomiting (7 cases, 15.9%), hepatosplenomegaly (5 cases, 11.4%), weight loss (3 cases, 6.8%), fatigue (3 cases, 6.8%), arthralgia (2 cases, 4.5%), and headache (1 case, 2.3%).

### 3.3 Laboratory findings

At the initial assessment, the peripheral complete blood count indicated white blood cell (WBC) counts between (1.17–5.98) × 10^9^/L, with 36 cases (81.8%) falling below 4.0 × 10^9^/L, differential counts revealed neutropenia (<1.5 × 10^9^/L) in 13 cases (29.5%), lymphopenia (<1.1 × 10^9^/L) in 9 cases (20.5%), and panleukopenia in 6 cases (13.6%). Reduced hemoglobin was observed in 25 patients (56.8%), and thrombocytopenia (69 × 10^9^/L) was identified in 1 case (2.3%). Elevated C-reactive protein (CRP) was seen in 12 cases (27.3%). The erythrocyte sedimentation rate (ESR) was measured in 41 patients (93.2%), with a mean of 32.7 ± 18.8 mm/h (range: 9–93 mm/h), and 35 cases (79.5%) exceeding 15 mm/h. Lactate dehydrogenase (LDH) levels, assessed in 43 patients (97.7%), varied from 228 to 931 U/L (mean ± SD: 495.5 ± 172.4 U/L), with elevations observed in 39 cases (88.6%). Alanine aminotransferase (ALT) levels, tested in all 44 cases, ranged from 8 to 142 U/L (mean ± SD: 38.5 ± 32.6 U/L), elevated in 10 patients (22.7%). Serum ferritin (SF) levels, tested in 38 patients (86.4%), ranged from 49.6 to 1,986 ng/ml (mean: 448.6 ± 448.9 ng/ml), elevated in 16 patients (36.4%). Immunoglobulin analysis of 44 patients showed elevated IgG in 8 (18.2%), IgM in 6 (13.6%), and IgA in 8 (18.2%). Cellular immune function testing in 30 patients (68.2%) revealed reduced CD4+ T-cell counts in 14 (31.8%) and decreased natural killer cell counts in 6 (13.6%). Among 41 tested cases (93.2%), autoantibodies were detected in 4 patients (9.1%) with low-to-moderate titers: Anti-SmD1 antibody positivity in 2 cases (4.5%), Anti-ribonucleoprotein (anti-RNP) antibody positivity in 1 case (2.3%), Anti-SSA/Ro antibody positivity in 1 case (2.3%). Rheumatoid factor (tested in 41, 93.2%) was mildly raised in 1 out of 41 tested individuals (2.3%). Complement testing (35 patients, 79.5%) showed mildly elevated C4 in 22 (50.0%) and elevated C3 in 4 (9.1%). Pathogen testing conducted on all 44 patients identified positive Mycoplasma pneumoniae IgM in 3 (6.8%), influenza B IgM in 2 (4.5%), and Epstein–Barr virus (EBV) capsid antigen IgM in 1 (2.3%) (EBV-DNA negative), and rhinovirus nucleic acid in 1 (2.3%). Coagulation function tests (all 44) showed mildly elevated D-dimer in 29 (65.9%). Two kids with secondary HLH exhibited higher levels of SF, LDH, and aspartate aminotransferase, alongside decreased fibrinogen and platelet counts; hemophagocytosis was observed in bone marrow examination (refer to Tables 1, 2). (see Table 1).

**Table 1:** Laboratory examination.

Indicators	Value	Range of data	*N* (**x%**)
WBC (×10^9^/L)	3.23 ± 0.86	<4	36 (81.8)
		>4	8 (18.2)
		Increased (>8)	12 (27.3)
CRP (mg/L)	9.05 ± 11.96	Normal (0–8)	32 (72.7)
ESR (mm/h)	32.7 ± 18.8	Increased (>15)	35 (79.5)
		Normal (0–15)	6 (13.6)
LDH (U/L)	495.5 ± 172.4	Increased (>300)	39 (88.6)
		Normal (0–300)	4 (9.3)
ALT (U/L)	38.5 ± 32.6	Increased (>40)	10 (22.7)
		Normal (0–40)	34 (77.3)
SF (ng/ml)	448.6 ± 448.9	Increased (>150)	16 (36.4)
		Normal (13–150)	22 (50.0)
ANA titer		Increased	4 (9.1)
		Normal (<1:80)	37 (84.1)

WBC, white blood cell; CRP, C reactive protein; ESR, erythrocyte sedimentation rate; LDH, lactate dehydrogenase; ALT, alanine aminotransferase; SF, ferritin; ANA, antinuclear antibody.

**Table 2:** Laboratory examination of two cases with secondary HLH.

Indicators	Case 1 (12 years and 9 months old)	Case 2 (12 years and 11 months old)
*Blood Routine*		
White Blood Cell (×10^9^/L)	1.17	1.5
Hemoglobin (g/L)	111	112
Platelet (×10^9^/L)	108	124
*Fibrinogen (g/L)*	2.861	1.47
*Biochemical Indicators*		
Lactate Dehydrogenase (IU/L)	863	802
Ferritin (ng/ml)	1,276.5	992.3
Triglyceride (mmol/L)	1.03	0.95
*Bone Marrow Cytology*	Bone marrow was hyperactive, with decreased granulocyte proliferation and numerous phagocytes visible.	The bone marrow showed active hyperplasia with vigorous proliferation of granulocytes, erythrocytes, and megakaryocytes, and phagocytes were observed.

### 3.4 Radiographic findings

**Figure 1:**
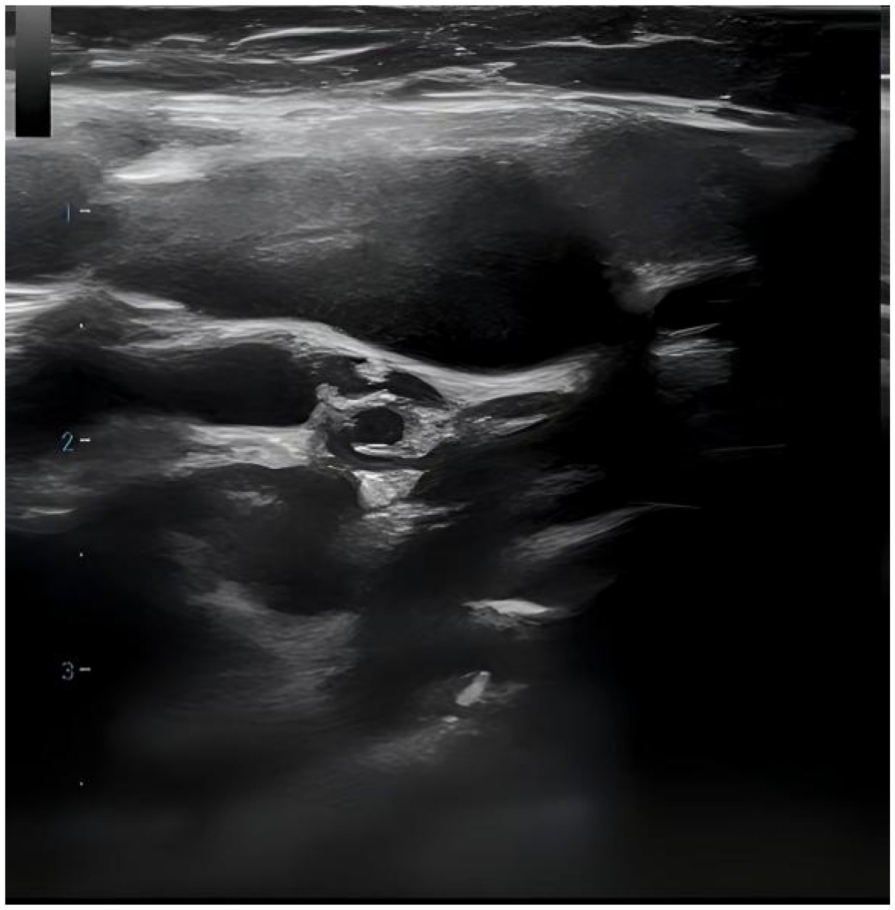
Ultrasonogram of lymph node.

**Figure 2:**
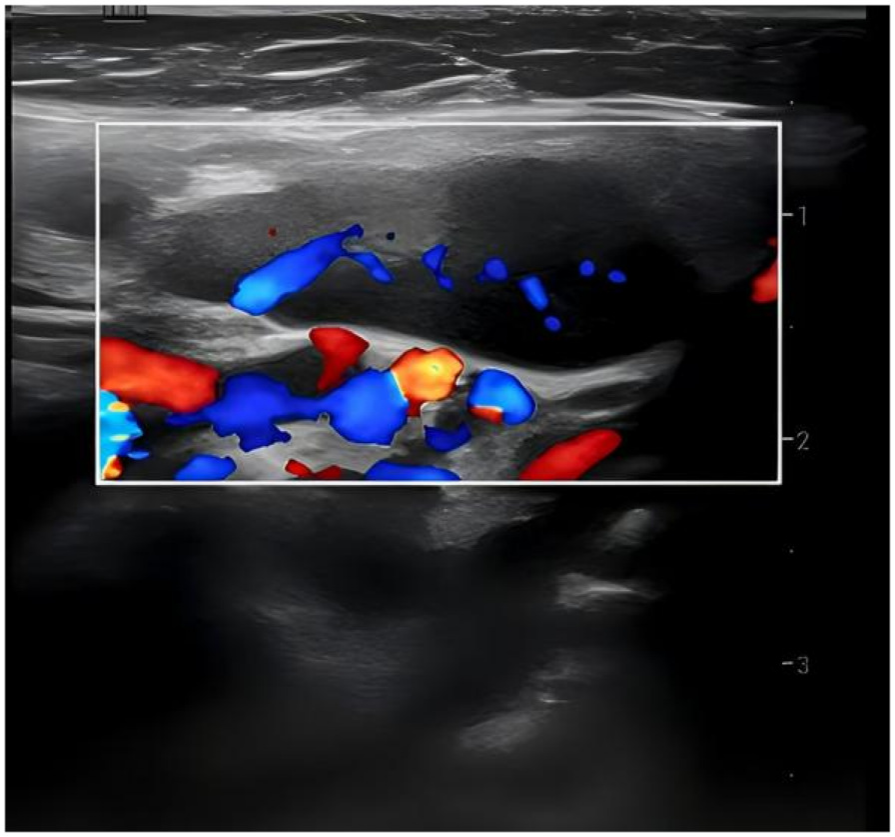
Rheography of lymph node.

**Figure 3:**
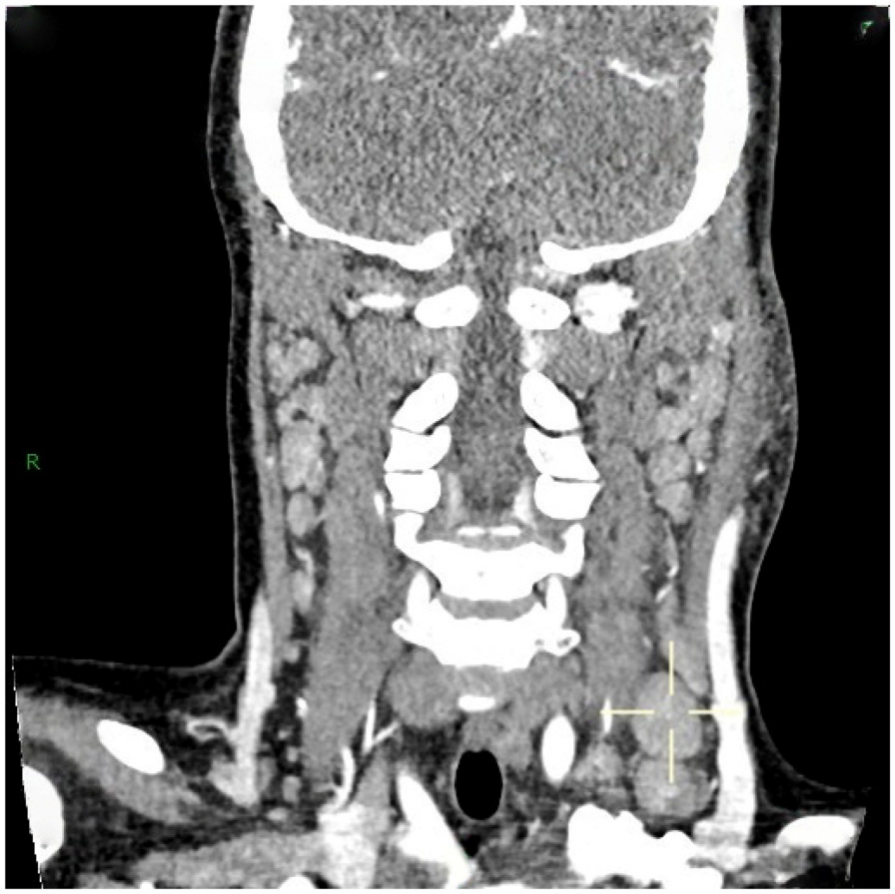
CT image of HNL lymph node.

**Figure 4:**
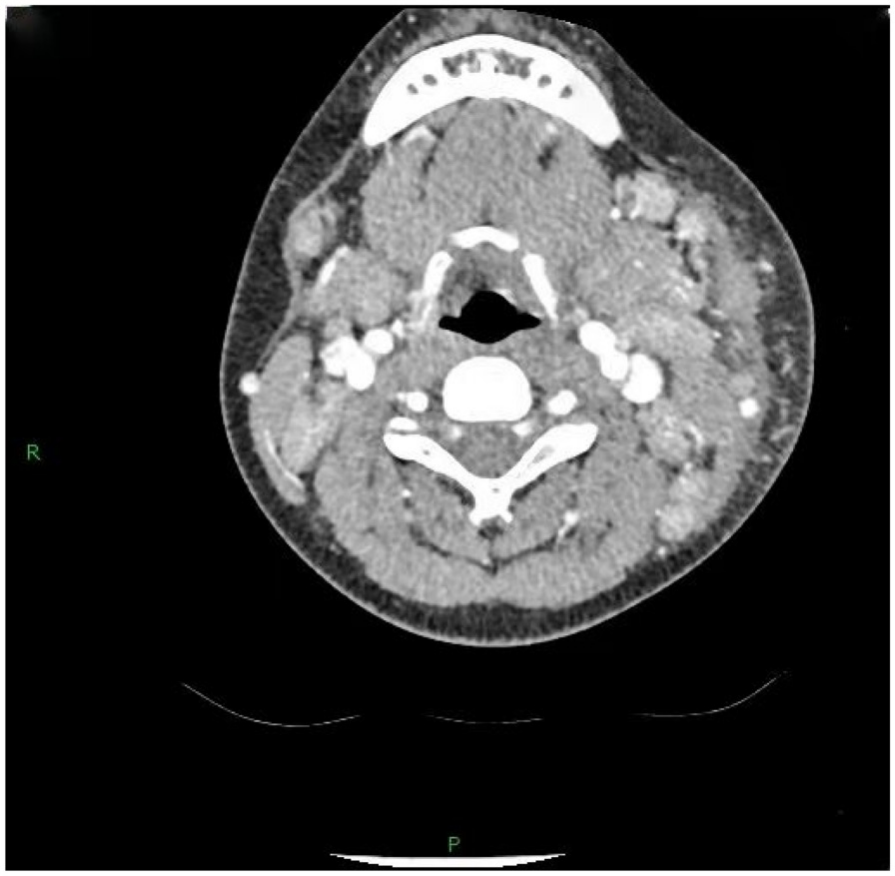
CT image of HNL lymph node.

36 out of 44 children (81.8%) underwent superficial lymph node ultrasonography, revealing subcutaneous hypoechoic nodules with distinct borders and regular morphology. Gray-scale ultrasound indicated a hyperechoic lymph node portal, devoid of calcification or necrosis. Doppler ultrasound demonstrated a normal blood flow pattern, with the portal vascular structure centrally located within the node (refer to Figures 1, 2). A CT examination of the neck was conducted in 7 cases (15.9%), indicating a nodular soft tissue density shadow with partial fusion and homogenous enhancement post-contrast (see Figures 3, 4). 38 out of 44 patients (86.4%) underwent thoracic/abdominal imaging (CT/x-ray/ultrasonography), with deep nodal involvement confirmed in 5 cases (11.4%).

### 3.5 Pathological findings

**Figure 5:**
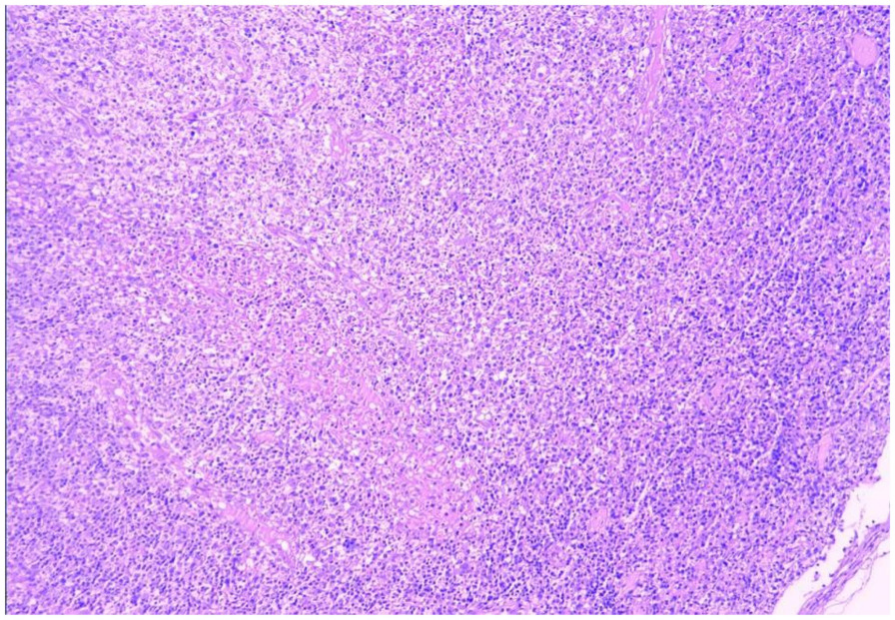
HNL lymph node pathology (H&E staining, 100× magnification, low-power microscopy reveals effacement of the lymph node architecture, replaced by patchy necrosis with surrounding prominent histiocytic proliferation).

**Figure 6:**
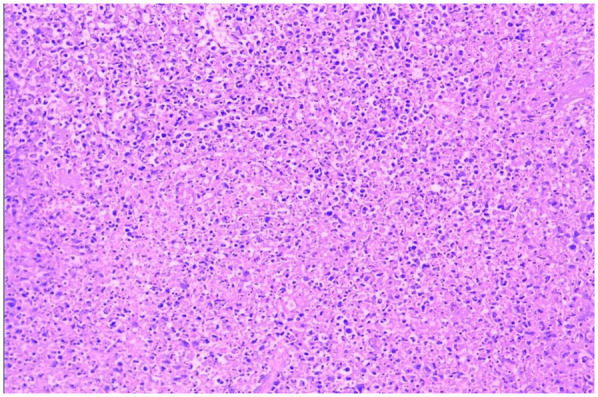
HNL lymph node pathology (H&E staining, 200× magnification, the necrotic foci are eosinophilic and amorphous, with abundant nuclear debris).

Bone marrow aspiration was performed in 30 patients, revealing hypercellular marrow in 16 cases (36.4%), hemophagocytosis in 2 (4.5%), myelodysplasia in 1 (2.3%), and normal findings in 11 (25.0%). All 44 patients underwent complete surgical excision of one affected lymph node. The largest cervical lymph node (levels II/III) was preferentially resected unless extra-cervical involvement was dominant, in which case the most metabolically active node (identified by imaging) was selected. the results showed that the lymph nodes had been severely damaged architecturally, with reactive histiocytic hyperplasia and widespread coagulative necrosis in both the cortical and paracortical regions. Necrotic foci contained abundant karyorrhectic debris, karyolysis and pyknotic nuclei, with no evidence of neutrophil infiltration. Immunohistochemical analysis confirmed CD68 positivity (see Figures 5, 6), corroborating a definitive diagnosis of HNL.

### 3.6 Treatment and outcomes

Prior to definitive diagnosis of HNL, 9 patients (20.5%) received empirical antibiotic therapy without clinical improvement. Spontaneous remission of fever and lymphadenopathy was observed in 8 cases (18.2%) without intervention. Glucocorticoids were administered to 35 patients (79.5%), of whom 27 (61.4%) received monotherapy with at an initial prednisone dosage of 0.5–1.5 mg/kg/day for 8–12 weeks. Two patients (4.5%) who developed secondary HLH received specific therapy: one underwent methylprednisolone sodium succinate pulse therapy (16 mg/kg/day for 3 days) followed by oral glucocorticoids combined with hydroxychloroquine (discontinued after 2 years), while the other was treated with intravenous immunoglobulin (0.5 g/kg/day for 2 days) followed by oral glucocorticoids for 6 months. Both secondary HLH patients remained stable during the dose tapering and discontinuation. Four patients (9.1%) received glucocorticoids in conjunction with naproxen for 16–20 weeks. Two patients (4.5%) were treated with glucocorticoids and methotrexate, with glucocorticoids administered for approximately 6 months and methotrexate continued for one year. One patient (2.3%) remained recurrence-free after cessation of 12 weeks of diclofenac monotherapy, with no recurrence observed post-discontinuation.

Follow-up duration ranged from 2 months to 6 years. During the period, recurrence was observed in 5 patients (11.4%): 4 (9.1%) experienced a single relapse, while 1 (2.3%) had two recurrences. All other cases exhibited favorable outcomes, with no progression to other rheumatic diseases.

## 4 Discussion

Histiocytic necrotizing lymphadenitis (HNL) can occur in individuals of any age, ethnicity, or gender; however, it is more prevalent in Asian populations, with an indeterminate incidence rate (4). Previous studies indicated a a greater prevalence among young females, while recent research reveals a broader age spectrum (19 months to 75 years) and a comparable gender distribution. To date, the etiology of HNL remains undetermined but may be associated with infectious factors, autoimmune factors, and genetic predisposition. Several studies have shown that multiple infectious agents, including EBV, CMV, P19, HIV, and types 6 and 8 of human herpesvirus, may trigger HNL (5–8). Nevertheless, no particular pathogen was associated with HNL in a Chinese investigation of 153 children (9). Even though every child in our cohort had extensive pathogen testing, we were unable to find evidence of a causal relationship.

The immunological etiology of HNL involves Fas-FasL-mediated apoptosis driven by cytotoxic CD8^+^ T lymphocytes, supported by histiocytic phagocytosis (10). Clinical manifestations primarily consist of nonspecific fever and lymphadenopathy. Studies indicates (11) that some patients initially present with unexplained fever, defined by low-grade or recurrent high-grade fever, an irregular fever pattern, with low-grade fever being the most prevalent, and a variable duration spanning from one week to several months. In 60%–90% of HNL patients, localized superficial lymphadenopathy is the primary symptom, with cervical lymphadenopathy being the predominant manifestation. This is often associated with the enlargement of other superficial lymph nodes such as axillary, and/or supraclavicular, inguinal regions, whereas generalized lymphadenopathy is rarely observed. The affected lymph nodes typically measure 5–40 mm in diameter (>60 mm is uncommon), with a soft consistency, well-defined borders, and good mobility, and may exhibit tenderness (12). No statistically significant difference in lymph node enlargement was seen between the pediatric and adult cohorts. All children in our cohort exhibited fever and superficial lymphadenopathy at onset, aligning with the clinical manifestations of HNL. Some patients may develop nonspecific rashes of varying morphologies, such as papules, erythema multiforme, urticaria, or drug eruptions. Among our cases, 10 patients (22.7%) exhibited rashes, predominantly manifesting as urticaria, papules, or erythema multiforme. Additionally, HNL patients may experience systemic atypical symptoms, including nausea, vomiting, diarrhea, weight loss, night sweats, anorexia, arthralgia, and myalgia (13). A minority of cases may progress to recurrent multi-organ involvement or evolve into other rheumatic immune diseases (14). Reports suggest a complex relationship between HNL and systemic lupus erythematosus (SLE), where SLE may precede, coincide with, or follow the onset of HNL (15). Therefore, children diagnosed with HNL necessitate long-term monitoring of autoantibody profiles and careful observation for multisystem involvement or advancement to other rheumatic immunological diseases. In our cohort, no cases progressed to other rheumatic diseases, potentially due to the limited sample size and relatively short follow-up duration for certain patients.secondary HLH, a severe complication of HNL, frequently occurs as a consequence of infections, rheumatic immune disorders, or malignancies. The pathophysiology is associated with cytokine storms driven by interleukin-6 (IL-6), interferon-γ (IFN-γ), and mutations in the perforin gene. Studies suggest that significantly elevated serum LDH levels may serve as a biomarker for HNL complicated by secondary HLH (16). Utilizing the 2016 EULAR/ACR/PRINTO classification criteria for macrophage activation syndrome (MAS) (17), both secondary HLH cases in our cohort exhibited remittent fever lasting over two weeks, alongside elevated serum ferritin (SF), lactate dehydrogenase (LDH), aspartate aminotransferase (AST), hypofibrinogenemia, thrombocytopenia, and hemophagocytosis observed in bone marrow analysis, thereby substantiating the diagnosis of HNL complicated by secondary HLH. A minimal percentage of HNL patients may develop complications such as aseptic meningitis, peripheral neuropathy, cerebellar ataxia, or autoimmune hepatitis (4, 18).

Studies indicate that HNL lacks disease-specific laboratory indicators. Hematological findings may include leukopenia, normal leukocyte counts, or leukocytosis, along with reduced hemoglobin, thrombocytopenia, increased lymphocyte proportion, and raised erythrocyte sedimentation rate (ESR). Non-specific abnormalities such as elevated CRP, SF, LDH, and transaminases are also observed (19). In our cohort, elevated ESR, SF, and LDH, as well as leukopenia (reduced white blood cell count) and anemia (low hemoglobin), were frequently identified, aligning with the existing literature.

Ultrasonography is a non-invasive, radiation-free procedure that verifies the dimensions of swollen lymph nodes and offers a differential diagnosis based on imaging characteristics (20). Som classification is an imaging-based technique for the categorization of cervical lymph nodes, offering accuracy and reproducibility in lymph node location. Regrettably, the anatomical positioning of the affected enlarged cervical lymph nodes was not meticulously delineated in this investigation in accordance with Som's classification (21). In this investigation, twenty-two cases (50.0%) had bilateral cervical lymph node expansion, in contrast to the more prevalent unilateral cervical lymph node enlargement often observed in the common progression of the disease (22–26). The subsequent indicators are regarded as characteristic ultrasonography manifestations of HNL (27): (1) Lesion location: mostly affects the posterior cervical area. (2) Gray-scale ultrasonography reveals hyperechoic lymph node gates, typically devoid of interior calcification, with necrotic foci being infrequent. (3) Doppler ultrasound: it reveals a normal blood flow pattern, centrally located vascular structures in the hilar region, and radial branching of arteries from the hilar region in both longitudinal and transverse perspectives. Lymphoma and reactive hyperplasia of lymph nodes may also exhibit centralized portal vascular structures and normal blood flow patterns (28). Nonetheless, both diseases typically do not exhibit evidence of perinodal echogenic amplification. Lymphomatous lymph nodes typically exhibit increased size, rounded morphology, heightened hyperechogenicity on grayscale imaging, and lack hilar echogenicity in contrast to hyperplastic lymph nodes (28). Moreover, lymphomatous lymph nodes typically exhibit a heterogeneous flow pattern (28, 29), whereas the majority of HNL lymph nodes demonstrate a normal hilar flow signal on energy Doppler. Significant necrosis in HNL lymph nodes necessitates differentiation from metastatic lymphadenitis and tuberculous lymphadenitis (30). Metastatic lymph nodes typically lack hilar echoes and are bigger and more spherical compared to those in HNL illness (30, 31). Moreover, metastatic lymph nodes typically exhibit peripheral blood flow with displaced portal arteries (28, 29, 32), while peri-lymph node echo amplification is rather rare. Tuberculous lymphadenitis typically exhibits no hilar echogenicity (28, 31) or perinodal echo amplification; nonetheless, calcifications are frequently observed, and hilar arteries may be misplaced or missing (28, 30). Consequently, perinodal echo enhancement appears to represent a distinctive expression of HNL. In this investigation, 36 cases (81.8%) underwent lymph node ultrasonography, and the results aligned with the characteristic ultrasound indicators of HNL.

Computed tomography (CT) of the neck serves as a significant reference prior to lymph node biopsy, and in the majority of individuals with HNL, the primary manifestations observed on CT are (25): (1) Characteristics of lymph node distribution: unilateral involvement is prominent, primarily occurring in the cervical lymph nodes of zones II-V. (2) Enhancement attributes: uniform augmentation in dimensions and uniform enhancement in enhancement. (3) Peripheral tissue alterations: approximately 81% of patients had evidence of peripheral lymph node involvement. This study involved 7 cases (15.9%) that received CT scan of the neck, revealing nodular soft-tissue density shadows, partial fusion, homogenous enhancement post-contrast, and an absence of necrotic indications. Necrotic symptoms necessitate suspicion of lymph node TB infection. The primary distinctions between HNL and TB infection encompass (33): (1) Lack of necrotic lymphadenopathy. (2) Anomalous cortical attenuation values of lymph nodes. (3) The CT value ratio of lymph nodes to surrounding muscles possesses discriminating significance. Kwon et al. identified CT as the preferred modality for assessing perinodal infiltration and substantial necrosis in lymph nodes in HNL. However, CT entails radiation dangers (34, 35), but ultrasound is noninvasive, safe, and more economical (29). Furthermore, due to its provision of real-time guidance, ultrasound-guided puncture biopsy can be executed when pathological confirmation of lymph node lesions is necessary. Kim et al. (36) report that ultrasound-guided puncture biopsy of cervical lymph nodes has a sensitivity of 97.9% and a specificity of 99.1%. Furthermore, ultrasound is superior in evaluating necrosis and can depict portal blood flow by energy Doppler imaging (28).

The clinical manifestations of HNL are nonspecific, and pathological examination of lymph node biopsies serves as the definitive diagnostic criterion for this disease. Pathological findings primarily consist of abundant apoptotic cells localized in the paracortical regions of the afflicted lymph nodes, surrounded by infiltrates of small lymphocytes, histiocytes, transformed lymphocytes, and plasmacytoid monocytes, with a complete absence of neutrophils. Coagulative necrosis with extensive karyorrhectic debris characterizes HNL, though distinct necrotic foci may be absent in the early stages of the disease. A critical immunohistochemical feature is the presence of clustered or small focal clustered or nodular aggregates of CD68-positive cells, with immunohistochemical staining revealing mixed proliferation of T cells and B cells (37). From a purely histopathological perspective, HNL exhibits three cardinal features that distinguish it from lymphoma: ① Architectural Preservation vs. Destruction:HNL maintains residual nodal architecture with focal paracortical expansion, whereas lymphomas typically efface nodal structures through diffuse proliferation of atypical lymphocytes (38). ② Cytological Composition: HNL shows a triad of: CD68+ histiocytes with crescentic nuclei and karyorrhectic debris, CD123+ plasmacytoid dendritic cells, Predominantly CD8+ T-cell infiltrates (39). In contrast, lymphomas demonstrate monomorphic proliferation of malignant lymphocytes (e.g., CD20+ B-cells in DLBCL) with high Ki-67 index (>90% vs. HNL's < 30%) (40). ③ Microenvironmental Patterns:HNL exhibits geographic necrosis surrounded by foamy histiocytes without angioinvasion. Lymphomas often show:Cohesive tumor sheets with stromal desmoplasia (e.g., Hodgkin lymphoma) and Vascular invasion (41). These features underscore the importance of immunohistochemical profiling (CD68/CD123/CD3/CD20/Ki-67) in equivocal cases. All 44 cases in this cohort underwent lymph node biopsy with pathological examination, confirming the diagnosis of HNL and eliminating malignant disorders such as lymphoma.

HNL must be distinguished from the following conditions: ① Lymph node tuberculosis infection: predominantly chronic in nature, presenting with symptoms indicative of tuberculosis infection, including low-grade fever, lethargy, and malaise; lymph nodes are typically painless in the initial stages, potentially becoming tender upon palpation in later stages; PPD and *γ*-interferon release tests frequently yield positive results; pathology is marked by caseous necrosis, with possible positive antacid staining. In this investigation, all cases tested negative for PPD and *γ*-interferon release tests, and the pathological examination did not indicate tuberculosis infection of the lymph nodes. ② Lymphoma: a chronic and progressive condition, frequently associated with low-grade fever, nocturnal diaphoresis, weight reduction, and painless lymphadenopathy, which may occur in any anatomical region. The pathology is marked by the monoclonal proliferation of lymphoid cells, with immunohistochemistry revealing specific lymphocyte markers (CD20, CD3, CD30, etc.). In this cohort of instances, the enlarged lymph nodes exhibited tenderness, which, when considered alongside the pathological evaluation of the lymph nodes, was incongruent. ③ EBV infection: primarily characterized by fever, pharyngitis, lymphadenopathy, hepatosplenomegaly, rash, among other symptoms. All children in this cohort tested negative for EBV-IgM/VCA-IgM, had low levels of heterogeneous lymphocytes, and no virus-infected cells were observed in the pathological examination, indicating incompatibility. Research indicates (11) that the misdiagnosis rate in children surpasses that of adults, partly due to insufficient awareness of the disease. Additionally, parental perceptions of lymph node biopsy as a traumatic procedure may lead to temporary refusals, consequently delaying diagnosis.

Currently, there are no standardized treatment guidelines for HNL. Conventional antibiotic therapy frequently proves futile, as the disease exhibits a self-limiting nature, with most symptoms and signs resolving spontaneously within 4 months. In this study, antibiotic therapy was ineffective in 9 patients (20.5%), while fever and lymphadenopathy healed spontaneously in 8 cases (18.2%), consistent with previous reports (6). Pharmacological intervention is necessary for patients with persistent symptoms, multisystem involvement, or extranodal lesions. Glucocorticoids and hydroxychloroquine have been identified as effective therapies for HNL (42–44), while intravenous immunoglobulin (IVIG) demonstrates efficacy in severe cases. Most patients experience rapid alleviation of symptoms following glucocorticoid therapy. In our cohort, 27 patients (61.4%) received glucocorticoid monotherapy, achieving normal body temperature within 24 h of treatment initiation and variable improvement in lymph node swelling and tenderness, further validating the therapeutic efficacy of glucocorticoids. Both secondary HLH cases had substantial clinical improvement with glucocorticoids or intravenous immunoglobulin (IVIG), which aligns with previous studies. Additionally, studies report that hydroxychloroquine monotherapy or its combination with glucocorticoids may be effective for managing recurrent or glucocorticoid-resistant HNL (45, 46). Nonsteroidal anti-inflammatory drugs (NSAIDs) can be used to alleviate fever and lymph node tenderness. However, the efficacy of ciprofloxacin, ofloxacin, and minocycline in in the treatment of HNL is not well-supported by robust evidence and requires further investigation (47–49).

The reported recurrence rate of HNL is approximately 3%–4%, with a mortality rate of 2.1% (4). In our cohort of 44 cases, follow-up durations ranged from 2 months to 6 years, during which 5 patients (11.4%) experienced recurrence—1 case (2.3%) relapsed twice, and 4 cases (9.1%) relapsed once—with no fatalities reported. Evidence suggests that a positive antinuclear antibody (ANA) is a high-risk factor for disease recurrence (50). Furthermore, leukopenia has been reported to be significantly associated with HNL recurrence (51). All 5 recurrent cases (11.4%) in our study exhibited leukopenia, consistent with previous findings. Research conducted by Stéphan Atarashi et al. (52) indicates a significant association between viral infections and the recurrence of HNL. Additionally, pancytopenia (deficiency of all three blood cell lineages) at disease onset has been identified by some scholars as a potential high-risk factor for unfavorable prognosis (53).

This study aimed to objectively examine histiocytic necrotizing lymphadenitis in children; nevertheless, the following limitations must be recognized. The study was a retrospective analysis conducted at a single center, with data obtained through the examination of hospital records. Moreover, the sample size of this investigation was comparatively limited, thereby diminishing the statistical power and impacting the reliability of the results. Despite our efforts to enhance the sample size, the final cohort remained comparatively small due to constraints in research funding and restricted data availability. Future extensive, multicenter collaborative prospective research will address this resource issue and yield more conclusive results.

In summary, clinicians should contemplate the potential of HNL in cases of unexplained fever with superficial lymphadenopathy and leukopenia that do not respond to standard antibiotic therapy. Early lymph node biopsy is essential for definitive diagnosis, thereby preventing superfluous diagnostic interventions and the misuse of medications. Disease management emphasizes symptomatic treatment, while glucocorticoids, hydroxychloroquine, or intravenous immunoglobulin are advised for individuals with persistent symptoms, multisystem involvement, or extranodal lesions. Given the risks of recurrence and progression to rheumatic immune diseases, long-term follow-up is essential.

## Data Availability

The raw data supporting the conclusions of this article will be made available by the authors, without undue reservation.
